# Pulmonary exposure to carbon black by inhalation or instillation in pregnant mice: Effects on liver DNA strand breaks in dams and offspring

**DOI:** 10.3109/17435390.2011.587902

**Published:** 2012-06-08

**Authors:** Petra Jackson, Karin Sørig Hougaard, Anne Mette Z. Boisen, Nicklas Raun Jacobsen, Keld Alstrup Jensen, Peter Møller, Gunnar Brunborg, Kristine Bjerve Gutzkow, Ole Andersen, Steffen Loft, Ulla Vogel, Håkan Wallin

**Affiliations:** 1 National Research Centre for the Working Environment, Copenhagen, Denmark; 2 Department of Science, Systems and Models, Roskilde University, Roskilde, Denmark; 3 Department of Public Health, University of Copenhagen, Copenhagen, Denmark; 4 Department of Chemical Toxicology, Division of Environmental Medicine, Norwegian Institute of Public Health, Oslo, Norway; 5 National Food Institute, Technical University of Denmark, Mørkhøj, Denmark

**Keywords:** Carbon black, nanoparticles, genotoxicity, inflammation, pulmonary exposure, in utero exposure, gestation and lactation

## Abstract

Effects of maternal pulmonary exposure to carbon black (Printex 90) on gestation, lactation and DNA strand breaks were evaluated. Time-mated C57BL/6BomTac mice were exposed by inhalation to 42 mg/m^3^ Printex 90 for 1 h/day on gestation days (GD) 8-18, or by four intratracheal instillations on GD 7, 10, 15 and 18, with total doses of 11, 54 and 268 (μg/animal. Dams were monitored until weaning and some offspring until adolescence. Inflammation was assessed in maternal bronchoalveolar lavage (BAL) 3-5 days after exposure, and at weaning. Levels of DNA strand breaks were assessed in maternal BAL cells and liver, and in offspring liver. Persistent lung inflammation was observed in exposed mothers. Inhalation exposure induced more DNA strand breaks in the liver of mothers and their offspring, whereas intratracheal instillation did not. Neither inhalation nor instillation affected gestation and lactation. Maternal inhalation exposure to Printex 90-induced liver DNA damage in the mothers and the *in utero* exposed offspring.

## Introduction

The need for risk assessment and an understanding of the toxicity of particles in ambient air and engineered nanoparticles is becoming more evident. It is concerning that some nanoparticles have the ability to induce DNA damage (Borm et al. 2004; Knaapen et al. 2004; Brauner et al. 2007; Schins and Knaapen 2007; Jacobsen et al. 2009; Moller et al. 2010). The primary genotoxicity of nanoparticles is related to their ability to induce reactive oxygen species (ROS) (Jacobsen et al. 2008b). It is less likely that insoluble nanomaterials such as Printex 90 cause DNA damage, because they only contain minute amounts of organic compounds and transition metals (Jacobsen et al. 2008a). Particles can however induce inflammation and thereby mediate secondary genotoxicity (Knaapen et al. 2004). Human exposure to ultrafine particles in the ambient air has been associated with increased risk of lung cancer, allergy, pulmonary and cardiovascular disease (Delfino et al. 2005; Pope and Dockery 2006; Weichenthal et al. 2007; Brunekreef et al. 2009; Krewski et al. 2009). Organisms under development may display increased sensitivity to nanoparticle toxicity. During development, frequent cell divisions allow only a short time for repair of DNA damage and the immune system is not fully functional. Early-life exposure might therefore predispose to cancer and other diseases later in life (Barton et al. 2005).

Little is known of potential health effects of nanoparticle exposure during fetal life and postnatal development. Epidemiological evidence indicates that environmental air pollutants, including fine particles, are associated with adverse pregnancy outcomes, such as premature birth, reduced birth weight, stillbirth, and postnatal respiratory deaths (Dejmek et al. 1999; Lacasana et al. 2005; Šrám et al. 2005; Slámma et al. 2007; Brauer et al. 2008; Pope et al. 2010). Maternal exposure to air pollution during pregnancy has been associated with increased levels of bulky DNA adducts and micronuclei in umbilical blood of new-borns (Pedersen et al. 2009). Adverse effects of diesel exhaust particles and titania-based nanoparticles in mothers and their offspring have been reported in a few animal studies (Reliene et al. 2005; Hougaard et al. 2008, 2010). It has been suggested that the fetus could be affected either: (1) Directly by particle translocation through the placenta; (2) by altered placental function; or (3) indirectly by circulating cytokines or other secondary messengers from an inflammatory process in the mother (Hougaard et al. 2011).

Soot from most combustion sources, such as diesel exhaust soot, partly consists of a carbonaceous core and inorganic and organic compounds, e.g., polycy-clic aromatic hydrocarbons (Utsunomiya et al. 2004). Printex 90 is a well characterized carbonaceous core particle that has been used extensively as a benchmark and as a model for diesel emission particles without adhered chemicals and metals. Some chemical and physical features are similar to other engineered carbon-based nanoparticles, e.g., single and multi-wall carbon nanotubes and C_60_ fullerenes that are handled in workplaces and occur in consumer products. Printex 90 consists of carbon with less than 1% organic and inorganic impurities (Brown et al. 2000; Wilson et al. 2002; Borm et al. 2005; Jacobsen et al. 2007). Health effects reported after exposure to carbon black are therefore assumed to be caused by the insoluble particle core rather than by associated compounds. Carbon black nanoparticles possess an intrinsic potential to generate reactive oxygen species (Wilson et al. 2002; Jacobsen et al. 2007, 2010; Folkmann et al. 2009; Yang et al. 2009). It is well known that pulmonary exposure to carbon black by instillation or inhalation induces an inflammatory response *in vivo* in rats (Driscoll et al. 1997; Brown et al. 2000; Wilson et al. 2002; Gallagher et al. 2003; Renwick et al. 2004; Sager and Castranova 2009) as well as mice (Saber et al. 2005; Jacobsen et al. 2009; Totsuka et al. 2009; Hougaard et al. 2010). Carbon black is also reported to be mutagenic (Driscoll et al. 1996; Jacobsen et al. 2007, 2010; Totsuka et al. 2009) and it induces lung tumors in rats (Mohr et al. 2006). It is uncertain whether occupational exposure to carbon black is related to cancer risk (Puntoni et al. 2001; Morfeld and McCunney 2007; Sorahan and Harrington 2007; Ramanakumar et al. 2008), but carbon black has been classified by the International Agency for Research on Cancer (IARC) as possibly carcinogenic to humans (Baan et al. 2006).

The purpose of the present study was to assess the effect of maternal gestational exposure to pure carbon black nanoparticles on the development of the offspring exposed *in utero.* Pulmonary exposure to carbon black nanoparticles causes pulmonary inflammation and genotoxicity. Therefore, we examined the effect of maternal pulmonary exposure to Printex 90 on DNA damage in the exposed offspring, along with traditional gestational and litter parameters.

## Methods

**Figure 1:**
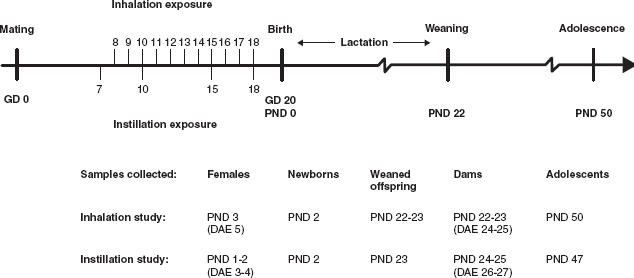
Experimental design. GD, gestation day (pregnancy day); PND, post natal day (days afterbirth); DAE, days after exposure. ‘Time-mated mice’ term was used for all exposed mice during gestation and when referring to results of ‘females’ and ‘dams’ together. Time-mated mice that had not given birth or had only few offspring were termed ‘females’.Time-mated females that gave birth were termed ‘dams’. PND 1 and PND 2 offspring were termed ‘newborns’. Offspring on PND 22-23 was termed ‘offspring at weaning’. Offspring on PND 50 (47) termed ‘adolescents’ had not reached sexual maturity. Time-mated mice were exposed by inhalation and intratracheal instillation to Printex 90. Time-mated mice inhaled 42 mg/m^3^ Printex 90 or filtered air for 1 hour/day for 11 consecutive days on GD 8-18. The daily dose would correspond to 12 hours at the Danish Occupational Exposure Limit of 3.5 mg/m^3^for carbon black. The total instilled doses were 0, 11, 54 and 268 (Xg/animal were distributed over four instillations on GD 7, 10, 15 and 18. The highest dose was chosen as to be similar to the estimated deposited dose in the pulmonary region from the inhalation study.

The study was comprised of two parts: An inhalation study and an instillation dose-effect study, with the highest dose being similar to the inhaled dose estimated to be deposited in the pulmonary region. The end points studied are classical gestational and lac-tational parameters. This was related to lung inflammation and DNA damage (see Figure 1 for details of the design and sampling point terminology).

### Animals

Time-mated, nulliparous adult female mice (C57BIV 6BomTac, Taconic Europe, Ejby, DK) were received on gestation day three (GD 3). The mice were immediately distributed in cages of five or six. Housing conditions have been described previously (Hougaard et al. 2008, 2010). On GD 4, mice were weighed and assigned to experimental groups. Body weight was also recorded before exposure on GD 7, and GD 10, 13, 15 (or 16) and 18.

All the procedures complied with the EC Directive 86/609/EEC and the Danish law regulating experiments on animals (The Danish Ministry of Justice, protection of experimental animals (Dyreforsogstilsy-net) with Animal Experiments Inspectorate Permission 2006/561-1123).

### Printex 90

The Printex 90 was a gift from Degussa-Hiils, Frankfurt, Germany (see Table I for previously published particle characterization).

**Table I:** Key physico-chemical characteristics of Printex 90.

Declared particle size	14 nm	Degussa-Hüls
Geometric mean size	65 nm (carbon spheres)	(Saber et al. 2005)
Morphology	Individual carbon black spheres mainly occurred in open-structured long chain aggregates and fewer large dense aggregates	(Saber et al. 2011)
Particle size distribution	The aggregates cover a wide size-range from <100 nm to 20–30 mm; the typical aggregate size is approximately 200 nm	(Saber et al. 2011)
Surface area	295–338 m^2^/g	(Saber et al. 2005) (Jacobsen et al. 2008b)
Pycnometric particle density	2.1 g/cm^3^	(Saber et al. 2005)
Chemical composition	99% C, 0.8% N and 0.01% H_2_	(Jacobsen et al. 2008b)
The total PAH content (Carbon black extract – Soxhlet)	0.0742 mg/g	(Jacobsen et al. 2007)
The total PAH content (DEP extract – NIST SRM 1650)	216 mg/g	(Jacobsen et al. 2008a)

PAH, polycyclic aromatic hydrocarbon (data included for comparison); DEP, diesel extract particles.

### Particle exposure

*Inhalation.* A total of 44 time-mated mice were exposed by whole-body inhalation exposure as described previously (Hougaard et al. 2008, 2010). Time-mated mice were placed in perforated steel-cage in a steel-framed pyrex glass exposure chamber. The animals were exposed whole-body to HEPA-filtered air or 42 mg/m^3^ aerosolized Printex 90 for 1 h per day from GD 8-18. Maximally 12 mice could be exposed at a time. Four groups of mice were exposed on each exposure day and the mice order was changed each time.

Printex 90 was fed into a small airstream by a rotating perforated disc micro-feeder (Fraunhofer-Institut fur Toxikologie und Experimentelle Medizin, Hannover, Germany) and it was dispersed into the nozzle with pressurized air (20 L/min; 5 bars). The mice were exposed at a slightly negative pressure in the exposure chamber between 07:30 and 14:30 h. The high dose-rate and short exposure time were chosen to avoid unnecessary stressing of the dams during gestation. We chose a relatively high dose because there are virtually no data on the developmental toxicity of nanoparticles. Still, the dose used (1 h exposure to 42 mg Printex 90/m^3^) corresponds to only one-and-a-half day exposure that Danish workers might experience at the time-weighted average occupational exposure limit (3.5 mg/m^3^ for carbon black) (The Danish Working Environment Authority 2007).

*Instillation.* The particle preparation and instillation procedures were described previously (Jackson et al. 2011). Printex 90 was sonicated for 8 min (10 s pulses and 10 s pauses, total sonication time 4 min) at a concentration of 1.675 mg/mL (67 jag/instillation) in 0.2 jam filtered, γ-irradiated Nanopure Diamond UV water (Pyrogens: < 0.001 EU/ml, Total Organic Carbon: < 3.0 ppb), using a 400 W Branson Sonifier S-450D (Branson Ultrasonics Corp., Danbury, CT, USA) mounted with a disruptor horn and operated at 10% amplitude. This dispersion was used for the high dose and diluted 1:5 for the medium dose (13.4 μg/instillation) and diluted futher 1:5 for the low dose (2.7 μg/instillation). Eighty time-mated mice were anesthetized with 3% Isofiurane and instilled with a vehicle or one of the three concentrations of Printex 90 dispersions (40 μL solution followed by 160 μL air) on GD 7, 10, 15 and 18. We chose to instill Printex 90 at times that would cover the major part of the fetal development. We tried to distribute the dose over that period assuming that a fraction of the particles would have been cleared rapidly, but that much of the dose would remain in the lungs for several weeks. Exposure took place between 08:30 and 14:30 h. Time-mated mice were instilled in different order each day, to reduce any variation that might be related to the time of exposure. The total instilled doses were 11, 54 and 268 μg/animal.

### Exposure control and characterization

*Inhalation.* Total aerosolized Printex 90 was sampled periodically from the exposure chamber using Milli-pore cassettes mounted with Millipore Fluoropore Filters (diameter 2.5 cm, pore size 0.45 μm). Filters were weighed immediately on a Sartorius Microscale (Type M3P). If needed, the airborne mass concentration was adjusted after the control measurement to the target concentration of 40 mg/m^3^.

The particle concentration-size-distribution was monitored on-line using a GRIMM Sequential (Stepping) Mobility Particle Sizer (Model No. 5.521) connected to a Condensation Particle Counter system (SMPS+C) and a GRIMM Dust Monitor (Model 1.105) for small (9.8-492.2 nm) and coarse particles (0.75-1.00 to >15 μm), respectively. The SMPS+C system was operated in fast scan mode *(ca.* 3 min and 40 s per spectrum) using correction for particle density and Stokes settling. The Dust Monitor collected data at a resolution of 6 s. The SMPS data were quality controlled omitting spectra collected during larger rapid concentration changes, which occurred during adjustments of exposure concentrations and results in false size distribution spectra.

*Instillation.* The particle size distribution in the Printex 90 dispersions was determined with a 633 nm He-Ne Dynamic Laser Scatter (DLS) Zetasizer nano ZS (Malvern Inc., UK). Data were analyzed using the Dispersion Technology Software (DTS) vs. 5.0 (Malvern Instruments Ltd). Samples were measured at 25° C in 1 mL disposable polystyrene cuvettes. For calculations of hydrodynamic size, we used the refractive (R_s_) and absorption indices (Rs) of 2.020 and 2.00, respectively, for Printex 90 and standard properties for H_2_O.

The dispersion of Printex 90 instillation fluid was also analyzed by Scanning Electron Microscopy (QUANTA 200 FEG MKII with EDX). Samples were prepared by placing one drop of the dispersions onto holey carbon-coated TEM Cu-grids (200 mesh) placed on filter paper in a Petri dish for quick absorption of liquid. The prepared Cu-filters were allowed to dry under a tilted lid in a HEPA-filtered LAF-bench (Microflow Advanced Biosafety Cabinet (ABS) Class II; now Bioquell Ltd, Hampshire, UK). Samples were transferred to polymer sample vials for storage as individual samples until analysis.

### Parturition and lactation

For terminology used see Figure 1. After the last exposure on GD 18, the time-mated mice were housed alone and monitored for birth. The expected day of delivery, GD 20, was assigned as post-natal day zero (PND 0) for the offspring. On PND 1, the offspring were counted and sex determined. Dams and newborns were weighed on PND 2 (inhalation study) and on PND 1 (instillation study). The remaining dams and offspring were also weighed on PND 8 (9), 12, 17 and at weaning on PND 22.

*Time-mated mice.* On PND 3 (5 days after the last inhalation exposure) and on PND 1-2 (3-4 days after the last instillation), the females were anesthetized with a mixture of Hypnorm-Dormicum and killed by withdrawal of heart blood. Bronchoalveolar lavage (BAL) fluid from each female was collected. The number of uterine implantation sites was determined; organs were dissected, placed in NUNC cryotubes, snap frozen in liquid N_2_ and stored at — 80° C until analysis. After weaning, at PND 22-23 (24-25 days after the last inhalation exposure) and PND 24-25 (26-27 days after the last instillation) the dams were killed and treated as described above for females.

*Offspring.* On PND 2, in the inhalation study, all except two male and two female offspring in each litter were removed and killed by decapitation. In the instillation study, one male and one female in each litter were removed and killed, leaving 3-5 offspring for further investigations. From the newborns, liver and lungs were dissected, placed in NUNC cryotubes, snap frozen in liquid N_2_ and stored at −80°C until analysis.

On PND 22, male and female offspring were randomly distributed into balanced experimental groups: A group for collection of organs at weaning PND 22-23, an adolescent group for maturation data and organs at PND 50 (47), and a group for behavioural testing and mating for a 2nd generation (to be published elsewhere). In the inhalation study all dissected organs were weighed. This included lungs and liver from the newborns; and lungs, liver, kidneys, spleen, heart and brain from the females, dams, offspring at weaning and adolescents. In the installation study only thymus of newborns and offspring at weaning was weighed, other organs were rapidly frozen to preserve the tissue quality. Relative organ weight was calculated as (organ weight/bodyweight)*100.

No time-mated mice or offspring died as a result of particle exposure. However, some time-mated mice were lost during the instillation study apparently due to other causes: One time-mated mouse assigned to the control group died before the start of the exposure; four time-mated mice from the low dose group and two time-mated mice from the medium dose group died during instillation. Data from these mice were excluded from the study. One control, one low dose, three medium dose, and five high dose dams were also lost due to spontaneous acute intestinal pseudo-obstruction, commonly observed in lactating C57B1/6 mice (described in Percy and Bart-hold 2001). Offspring of these dams were also killed immediately. Since the cause of this disease was related to lactation only, the gestation data and newborns data on PND 2 were included in the study.

### BAL preparation and analyses

BAL was collected under hypnorm-dormicum anaesthesia by washing lungs four times with 0.8 mL 0.9% sterile saline through the trachea. The BAL was immediately put on ice until BAL fluid and BAL cells were separated by centrifugation at 4°C and 400 *g* for 10 min. The BAL cells were re-suspended in 100 *\iL* medium (HAM F-12 with 10% fetal bovine serum and 1% penicillin-streptomycin). The number of macrophages, neutrophils, lymphocytes, eosinophils and epithelial cells were determined in 40 *\iL* re-suspension by counting 200 cells prepared and analyzed as described (Jackson et al. 2011). Counts are presented relative to the total cell number in the BAL fluid. The total number of living and dead cells in BAL samples was determined in further diluted suspension (20 uL cells in 180 uL HAM F12 medium with FBS and PS) by counting in a hemocytometer with trypan blue dye (inhalation study samples) or in a NucleoCounter (instillation study samples), following the standard kit procedure (Chemometec, Denmark).

The remaining re-suspension (40 JJL) was mixed with 160 JJL freezing medium (HAM F-12, 10% FBS, 1% PS, containing 10% DMSO) and stored at -80°C for later comet assay analysis.

### Detection of DNA strand breaks

The level of DNA strand breaks in frozen BAL and liver cells was determined by the alkaline comet assay as described in (Dybdahl et al. 2004; Bornholdt et al. 2007) based on a protocol by (McNamee et al. 2000). The strand breaks measured by the assay represent a mixture of direct strand breaks, alkaline labile sites and transient breaks in the DNA due to repair processes (Collins 2009). BAL cell suspensions in freezing medium with 10% DMSO were thawed quickly at 37°C. For liver, deep frozen samples *(ca.* 40 mg) were pressed through a metal stapler (diameter 0.5 cm, mesh size 0.4 mm) into Merchant's media (0.14 M NaCl, 1.47 mM KH_2_PO_4_, 2.7 mM KC1, 8.1 mM Na_2_HPO_4_, 10 mM NaEDTA, pH 7.4) for inhibition of endogenous DNA cleaving enzymes (Brunborg et al. 1996). Samples were rapidly embedded in agarose moulded onto a hydrophilic polyester film, a GelBond® film (Lonza Rockland Inc. ME, USA), which was then quickly immersed into lysing solution at 4°C. Samples were alkaline treated and subjected to alkaline electrophoresis (pH > 13) at 25 V and current of 292-296 mA for 20 min in circulating electrophoresis solution. The gels were fixed, and later stained with SYBR Gold fluorescent dye (Molecular probes, Denmark; 1:10,000) and 50 randomly selected comets were scored by fluorescent microscopy using Kinetics® image analysing system (version 3.9). DNA damage was quantified as %DNA intensity in the comet tail. Electrophoresis efficiency was validated by including identical H_2_O_2_ exposed A549 cells as positive controls. In independent experiments the absolute frequency of lesions (per million base pairs) was calibrated using ionizing radiation (Moller et al. 2004). The primary comet assay endpoints were recalculated to the number of lesions per million base pairs, assuming that one unit increase in the %DNA in the tail corresponds to 0.0554 lesions/10^6^bp.

Two different comet methods were used to process the inhalation and instillation samples. Inhalation samples were cast in polyethylene moulds with eight wells (well diameter 19.5 mm with 130 μL per sample). Four films with eight samples each were processed per electrophoresis. The instillation samples were analyzed using a high throughput protocol allowing 48 samples per GelBond® film, developed at Norwegian Institute of Public Health (Gunnar Brunborg and Kristine Bjerve Gutzkow) within the COMICS EU Project. Cell/agarose suspension was dripped with a multichannel pipette onto a GelBond^(R)^ film (7 μL per sample). Eight films were processed per electrophoresis, in two parallel electrophoresis tanks. Due to preparation time, the lysing procedure varied between 1-2 h for samples in the present study (up to 3.5 h). The high volume protocol allowed processing of all related samples on one film and reduced the variation caused by increased processing time and different electrophoreses.

The level of oxidatively damaged DNA in the liver from offspring of dams exposed to Printex 90 by inhalation was also assessed as formamidopyrimidine DNA glycosylase (FPG) (kindly donated by Andrew Collins, Oslo, Norway) enzyme sensitive sites (Folkmann et al. 2007). FPG sites were recalculated to lesions/10^6^bp by factor 0.0261.

To prepare liver samples for comet analysis, the inhalation liver samples were cut on dry ice. This procedure was later modifyed such that the samples were crushed in liquid N_2_, a method that gives results with smaller variation and reduced background. Instillation liver samples were cut from fresh livers; samples were immediately frozen and not handled until analysis. Strand breaks were reported as lesions/ 10^6^ bp for all experiments. However as the comet analyses were performed in different experiemetnal set-ups and because H_2_O_2_ exposed controls were used that do not allow direct estimation of the number of induced strand breaks, the levels of strand breaks may not be directly comparable between experiments. Consequently emphasis was put on the comparison within experiments.

### Data analyses

The accepted level of statistical significance was 0.05. Litter was considered the statistical unit. Gestational parameters were analyzed by Kruskal-Wallis One-Way Analysis of Variance. Weight data were analyzed by analyses of variance (ANOVA), with treatment as factor, and day of weighing (GD, PND) as repeated measure. Litter size was used as co-variable for weight data during gestation. The number of litters was compared by Fisher's exact test. Remaining data (BAL results, comet assay results, organ weights) were analyzed by analyses of variance (ANOVA), with treatment, day of sampling (PND) and sex (where relevant) as factors. Significant results from overall analyses were analyzed by pair wise comparisons. Data were analyzed separately for each day of sampling and instillation study results were further analyzed by dose in Fisher's Least-Significant-Difference Test. Females sampled 3-5 days after exposure and dams sampled after weaning were compared, even though the groups differed by timing of sampling and also by pregnancy status. The “female” group consisted of pregnant mice with small litters and non-pregnant mice. Pregnancy is reported to alter the level of inflammatory response (Fedulov et al. 2008; Lamoureux et al. 2010), thus different background levels had to be accepted. Analyses were performed on SYSTAT Software Package version 9 and Statistical Tables for PC users.

## Results

### Particle exposure

**Figure 2:**
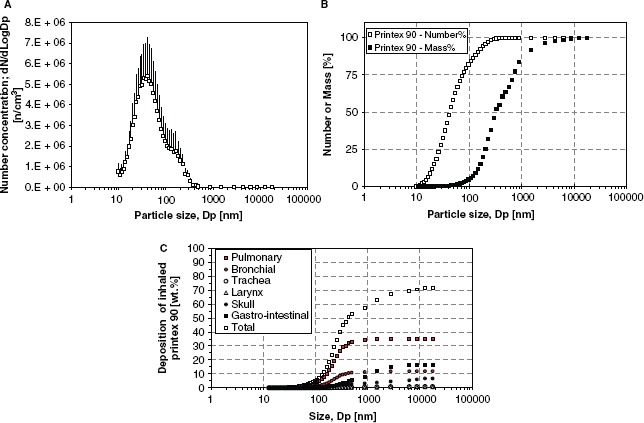
Size distribution data for the inhalation exposure and calculated deposition in mice. (A) Average number size distribution of Printex 90 dust in the inhalation exposure chamber. Error bars are the standard deviation of the different individual exposure runs. (B) Accumulated average number and mass distribution of particles in the exposure chamber. (C) The calculated accumulated particle deposition in the mice using a modified version of the model applied in Jacobsen et al. (2009).

*Inhalation.* Time-mated mice inhaled 42 mg/m^3^ (the variation of the dose between groups was 41.73 ± 0.01 mg/m^3^) Printex 90 or filtered air for 1 h/day for 11 consecutive days. This would correspond to 12 h at the Danish Occupational Exposure Limit of 3.5 mg/m^3^ for carbon black. The particle number concentration in the exposure atmosphere was 4.09 ± 0.03 × 10(6)/cm^3^. The average particle size-distribution was multimodal and highly dominated by sub-100 nm particles. The most abundant size number was in the order of 41 nm, which was also the average size (see Figures 2A and 2B). The average size by mass was 310 nm, and the mass size distribution was bimodal with one mode around 290 nm and a coarser mode at *ca.* 1.5 jam (see Figure 2B). Only 5% of the mass was below 100 nm, 83% of the particles were in this ultrafine size range by number.

Based on a deposition model revised from (Jacobsen et al. 2009), 34.8% of the particle mass was expected to deposit in the pulmonary region and 20.1% of particles were expected to deposit in the extra-pulmonary region (11.9% bronchial region, 0.9% trachea, 0.6% larynx, and 6.7% scull). An additional 16.6% was expected to deposit in the gastrointestinal tract. The calculated accumulated deposition pattern is presented in Figure 2C. The total inhaled dose was 826 jo,g Printex 90 (1 h/day × 11 days × 41.7 μg/dm^3^ × 1.8 dm^3^/h). Based on deposition estimates, the pulmonary dose would be 287 μg/animal; or 13.1 mg/kg, based on an average body weight of 22 g on GD 4 (287 μg/22 g). The corresponding inhaled particle surface area was at least 0.085 m^2^/animal (295 m^2^/g × 0.000287g/ani-mal); or 3.9 m^2^/kg (0.085 m^2^/0.022 kg) equal to 243 m^2^/kg lung (0.085 m^2^/0.00035 kg lung).

In addition to the particles that directly enter the gastrointestinal region, a contribution from the extra-pulmonary region was expected, because particles are removed from the lungs by the mucociliary escalator and are ultimately swallowed. Therefore, up to 36.7% (303 μg/animal) of Printex 90 nano-particles was expected to enter the animal via the gastrointestinal tract, increasing the final particle mass to 590 μg/animal; or 26.8 mg/kg. In the whole-body exposure used in the present study, particles were deposited on the fur and grooming can therefore be expected to increase the total dose even further.

**Figure 3:**
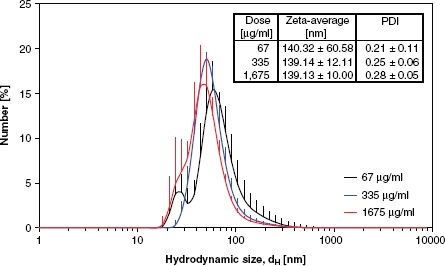
Hydrodynamic size distribution of intratracheally instilled Printex 90 dispersions. Particle size distribution at the three instillation concentrations for the intratracheal instillation exposure measured by Dynamic Light Scattering. Error bars show the standard deviation of six measurements. Inserted table shows the average intensity size and polydispersivity index.

**Figure 4:**
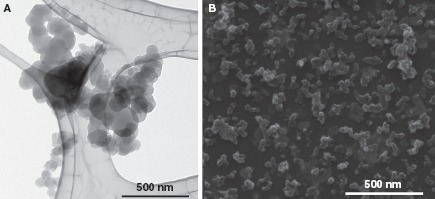
Printex 90 intratracheal instillation exposure characterization by TEM and SEM. (A) Transmission Electron Micrograph of agglomerated small- and medium size Printex 90 aggregates (67 Ug/mL). (B) Scanning Electron Micrograph illustrating the overall size and morphologies of Printex 90 aggregates and agglomerates in the dispersions (1.675 μg/mL).

*Instillation.* Time-mated mice were instilled four times during gestation on GD 7, 10, 15 and 18 with Printex 90 or vehicle. The instilled dose in the highest dose group 268 μg/animal, 12.2 mg/kg; 0.080 m^2^/animal; or 3.6 m^2^/kg. The dose was similar to the estimated dose deposited in the pulmonary region, calculated from the estimates in (Jacobsen et al. 2009). The final instillation doses in the lower dose groups were 54 and 11 jag/animal, respectively; 2.5 and 0.5 mg/kg; 0.016 and 0.003 m^2^/animal respectively; or 0.72 m^2^/kg and 0.15 m^2^/kg, respectively. The particle size distribution was similar in the three instilled dispersions with concentrations of 1675, 335 and 67 jag/mL (see Figure 3), and was stable for more than 1 h. The average zeta-size was approximately 140 nm and the hydrodynamic number size-distributions had a peak size between 50 and 60 nm (see Figure 3). When converted to volume-distributions, minor amounts of jam-size particles and two smaller size modes with peak sizes around 50-60 nm and 200-400 nm were identified. The observed DLS sizes were confirmed by TEM and SEM, with a wide size distribution of nm- to μm-size free and agglomerated particles. The agglomerates consisted of spherical to sub-spherical carbonaceous particles as well as minor amounts of free single primary spheres (see Figures 4A and 4B).

### Lung inflammation in the time-mated mice

Analysis of BAL fluid cell composition by differential cell count indicated the presence of inflammation in the lungs of time-mated mice exposed to Printex 90 both by inhalation and instillation (see Table II).

**Table II:** BAL cell composition of females or dams exposed to Printex 90 by inhalation or intratracheal instillation and control mice.

	Inhalation		Instillation			
		
	Control	Printex 90 41.7 mg/m^3^ ∼ 287 mg/animal	Control	Printex 90 11 mg/animal	Printex 90 54 mg/animal	Printex 90 268 mg/animal
Females 3–5 days after exposure						
Total count	66.07 ± 6.03	96.88 ± 10.72*	168.00 ± 45.03	175.50 ± 15.56	200.40 ± 19.22	292.00 ± 54.15
Dead cells	1.08 ± 0.36	0.79 ± 0.32	14.00 ± 7.21	12.50 ± 4.27	11.20 ± 5.24	18.67 ± 5.93
Macrophages	56.35 ± 4.76	69.21 ± 6.74	139.74 ± 42.31	128.79 ± 12.01	154.45 ± 20.45	115.51 ± 22.57
Neutrophils	1.01 ± 0.61	11.47 ± 3.06**	3.69 ± 2.08	5.50 ± 1.68	9.32 ± 4.75	105.75 ± 26.19***
Lymphocytes	0.76 ± 0.18	2.59 ± 0.63*	3.50 ± 0.95	7.18 ± 3.12	7.22 ± 1.52	27.03 ± 9.68**
Eosinophils	0.46 ± 0.31	3.97 ± 2.58	5.92 ± 3.66	22.24 ± 10.04	11.37 ± 6.37	33.04 ± 9.07
Epithelial cells	7.50 ± 1.28	9.63 ± 1.61	15.15 ± 4.22	11.80 ± 2.73	18.04 ± 3.43	10.67 ± 3.77
Dams at weaning 24–27 days after exposure						
Total count	44.08 ± 3.37	54.81 ± 4.26(*)	137.25 ± 5.64	93.43 ± 8.27	154.50 ± 10.80	361.29 ± 43.40***
Dead cells	0.68 ± 0.10	0.45 ± 0.11	6.88 ± 1.94	3.43 ± 2.26	4.50 ± 2.23	19.43 ± 2.75***
Macrophages	31.90 ± 3.25	37.73 ± 3.20	113.68 ± 4.91	82.98 ± 7.50	128.78 ± 9.50	131.79 ± 16.44
Neutrophils	0.41 ± 0.07	4.82 ± 1.07***	2.85 ± 0.90	0.90 ± 0.16	5.23 ± 1.65	173.26 ± 22.66***
Lymphocytes	1.35 ± 0.34	2.02 ± 0.50	7.71 ± 2.14	3.38 ± 0.67	6.94 ± 1.10	34.12 ± 8.38***
Eosinophils	0.37 ± 0.29	0.67 ± 0.47	2.26 ± 0.97	0.33 ± 0.16	2.84 ± 2.28	1.29 ± 0.42
Epithelial cells	10.03 ± 0.85	9.56 ± 0.80	10.76 ± 1.77	5.83 ± 1.18	10.71 ± 1.09	20.72 ± 4.53

BAL, bronchoalveolar lavage. Data presented as mean cell number × 10^3^ in BAL ± SEM. (*)*p* ∼ 0.05,

* *p* < 0.05,

** *p* < 0.01,

*** *p* < 0.001.

*Inhalation.* Time-mated mice exposed to Printex 90 by inhalation had more neutrophils in BAL fluid compared to their controls 5 and 24 days after exposure (5 days: 11.4-fold increase, *p* = 0.008; 24 days: 11.6-fold increase, *p* < 0.001). Females exposed to Printex 90 by inhalation had more lymphocytes in BAL fluid 5 days after exposure (3.4-fold increase, *p* = 0.020) and total cell counts were higher at both time-points (5 days: 1.5-fold increase, *p* = 0.032; 24 days: 1.2-fold increase, *p* = 0.057).

*Instillation.* Change in the counting method caused a sustained increase in all types of cells in BAL, except epithelial cells, when comparing the instillation and inhalation control groups. It is possible that some increase is also due to the instillation procedure, the contribution is however minor (Jackson et al. 2011). The time-mated mice exposed to Printex 90 by instillation also had more neutrophils in the BAL 3 and 26 days after exposure compared to the instilled control animals. This was statistically significant in the high dose group only (3 days: 28.7-fold increase, *p* < 0.001; 26 days: 60.9-fold increase, *p* < 0.001). Also, more lymphocytes were observed 5 and 26 days after exposure in the high dose group (3 days: 7.7-fold increase, *p* = 0.005; 26 days: 4.4-fold increase, *p* < 0.001). Printex 90 instilled dams had more total cell counts and more dead cells in BAL fluid 26 days after exposure in the high dose group (total cell count increased 2.6-fold, *p* < 0.001; dead cells increased 2.8-fold, *p*< 0.001).

### DNA strand breaks

DNA strand breaks were evaluated by the Comet assay in time-mated mice BAL cells and liver cells, and in liver cells of offspring (see Table III).

**Table III:** Level of DNA strand breaks for females, dams or offspring exposed to Printex 90 by inhalation or intratracheal instillation and control mice.

	Inhalation		Instillation			
		
	Control	Printex 90 41.7 mg/m^3^ ∼ 287 mg/animal	Control	Printex 90 11 mg/animal	Printex 90 54 mg/animal	Printex 90 268 mg/animal
Females 3–5 days after exposure						
BAL	0.55 ± 0.06	0.70 ± 0.10	0.90 ± 0.04	0.91 ± 0.09	0.78 ± 0.07	0.72 ± 0.07
Liver	2.57 ± 0.19	3.25 ± 0.21*	0.66 ± 0.06	0.55 ± 0.07	0.57 ± 0.07	0.52 ± 0.07
Dams at weaning 24–27 days after exposure						
BAL	0.58 ± 0.05	0.60 ± 0.04	0.62 ± 0.02	0.69 ± 0.06	0.58 ± 0.01	0.50 ± 0.03**
Liver	1.24 ± 0.07	1.94 ± 0.11***	0.52 ± 0.05	0.55 ± 0.09	0.48 ± 0.02	0.58 ± 0.04
Offspring						
Liver newborns PND 2	3.50 ± 0.28	3.64 ± 0.37	0.66 ± 0.02	0.65 ± 0.03	0.73 ± 0.02	0.64 ± 0.03
Liver offspring at weaning PND 22–23	1.13 ± 0.09	1.56 ± 0.08***	0.39 ± 0.03	0.41 ± 0.04	0.42 ± 0.04	0.42 ± 0.03
Liver adolescents PND 50 (47)	1.10 ± 0.16	1.69 ± 0.14**	0.54 ± 0.04	0.48 ± 0.04	0.47 ± 0.04	0.52 ± 0.03

BAL, bronchoalveolar lavage; PND, post natal day (days after birth). Data are presented as mean number of lesions per 106 base pairs ± SEM (calculated from %DNA results). Offspring data are calculated as litter average, when sibling liver tissues were analyzed at the same collection point.

* *p* < 0.05,

** *p* < 0.01,

*** *p* < 0.001.

### Inhalation

*Time-mated mice.* Inhalation of Printex 90 did not affect the level of DNA strand breaks in BAL fluid cells 5 and 24 days after exposure in the exposed time-mated mice compared to their controls *(p* = 0.20). Exposure induced higher levels of DNA strand breaks in the liver 5 and 24 days after exposure in the time-mated mice compared to their controls (5 days: 1.3-fold increase, £ = 0.04; 24 days: 1.6-fold increase, *p* < 0.001).

*Offspring.* In the offspring exposed to Printex 90 by maternal inhalation exposure, the level of DNA strand breaks was higher in offspring liver at weaning and in adolescents, compared to their controls (weaning: 1.4-fold increase, *p* = 0.001; adolescents: 1.5-fold increase, *p* = 0.011). Overall, newborns displayed higher levels of DNA strand breaks in liver tissues compared to tissues from the older offspring at weaning and from adolescents, both in the Printex 90 and the control group *(p* < 0.001). Each data point represents an average value of two separate comet assay runs.

The level of oxidatively generated DNA damage in the liver of offspring from the inhalation study was also determined by the level of formamidopyrimidine DNA glycosylase (FPG) enzyme sensitive sites. There was no consistent increase in oxidatively generated DNA damage in the offspring liver cells in newborns, at weaning or in adolescents (newborn exposed 0.91 ± 0.27 vs. control 0.71 ± 0.20; weaning exposed 1.05 ± 0.12 vs. control 1.28 ± 0.13; adolescents exposed 0.87 ± 0.10 vs. control 1.20 ± 0.11; two-way ANOVA *p* = 0.60; all data are presented as lesions per 10^6^ base pairs) (data not graphically shown).

### Instillation

*Time-mated mice.* Intratracheal instillation of Printex 90 did not affect the level of DNA strand breaks in BAL cells in the females *(p* = 0.30), while the dams exposed by instillation had significantly less DNA strand breaks in BAL cells in the high dose group 26 days after exposure compared to control dams (20% reduction, *p =* 0.007). No increase in the level of DNA strand breaks was observed in the liver of time-mated mice exposed to Printex 90 by instillation compared to their controls (two-way ANOVA *p* = 0.85). Each data point represents an average of duplicate scored in two separate rounds.

*Offspring.* In the offspring exposed to Printex 90 by maternal intratracheal instillation, the level of DNA strand breaks in liver cells was comparable to their controls *(p* = 0.8). Interestingly, as we also observed in the inhalation study, the level of DNA strand breaks was generally higher in liver cells from newborns, compared to tissues from older siblings at later time points *(p <* 0.001).

### Maternal and litter parameters

Gestational and litter parameters in exposed dams and their offspring were similar to controls both after inhalation or instillation of Printex 90 (weight gain during gestation and lactation, gestation length, offspring weight at birth, during lactation and maturation, litter size, gender ratio, number of implantations, and postnatal viability; see Table IV).

**Table IV:** Gestation, lactation and developmental parameters of dams and offspring exposed to Printex 90 by inhalation or intratracheal instillation and control mice.

	Inhalation		Instillation			
		
	Control	Printex 90 41.7 mg/m^3^ ∼ 287 mg/animal	Control	Printex 90 11 mg/animal	Printex 90 54 mg/animal	Printex 90 268 mg/animal
Time mated/exposure groups	22	22	24	17	17	22
Dam arrival weight, GD 4 (g)	22.21 ± 1.47	21.82 ± 1.27	23.01 ± 1.33	22.37 ± 1.20	22.59 ± 1.03	22.87 ± 0.89
Number of litters PND 1	18	17	20 (−1†)	10 (−4†)	11 (−2†)	20
Dam weight gain, GD 7–18 (g)‡	11.25 ± 0.58	10.64 ± 0.71	11.94 ± 0.33	12.00 ± 0.73	11.00 ± 0.61	10.79 ± 0.36
Dam lactation weight gain PND 1(2)–17 (g)	4.31 ± 0.27	4.34 ± 0.41	4.40 ± 0.43	4.38 ± 0.81	4.85 ± 0.45	4.93 ± 0.46
Gestation length (days)	19.89 ± 0.07	20.06 ± 0.06	20.00 ± 0.00	20.00 ± 0.00	20.09 ± 0.09	20.05 ± 0.05
Implantations	7.39 ± 0.56	7.59 ± 0.54	9.25 ± 0.21	9.27 ± 0.57	8.08 ± 0.62	8.32 ± 0.46
Implantation loss (%)	14.59 ± 3.88	17.01 ± 4.57	21.52 ± 2.52	30.74 ± 8.42	25.61 ± 7.75	24.16 ± 3.70
Live pups per litter PND 1	5.09 ± 0.91	4.95 ± 0.74	7.30 ± 0.34	7.00 ± 0.67	6.82 ± 0.52	6.20 ± 0.34
Offspring dead during lactation (%)	4.10 ± 1.98	1.72 ± 1.19	5.49 ± 2.06	10.00 ± 10.00	1.30 ± 1.30	3.26 ± 1.94
Birth weight females (g)	1.40 ± 0.04	1.41 ± 0.05	1.33 ± 0.03	1.30 ± 0.03	1.31 ± 0.04	1.30 ± 0.03
Birth weight males (g)	1.43 ± 0.03	1.42 ± 0.05	1.35 ± 0.02	1.38 ± 0.04	1.36 ± 0.04	1.35 ± 0.04
Weight gain females PND 1(2)–22 (g)	8.02 ± 0.23	7.66 ± 0.32	6.37 ± 0.29	7.34 ± 0.47	6.96 ± 0.45	7.50 ± 0.29
Weight gain males PND 1(2)–22 (g)	8.28 ± 0.18	7.89 ± 0.38	7.12 ± 0.32	7.33 ± 0.62	7.86 ± 0.60	7.86 ± 0.24
Sex ratio§	0.42 ± 0.06	0.51 ± 0.06	0.46 ± 0.04	0.66 ± 0.06	0.56 ± 0.06	0.45 ± 0.04

GD, gestation day (pregnancy day); PND, post natal day (days after birth). Dams were allowed to deliver their offspring on gestation day (GD) 20, equal to post natal day (PND) 0. Weights of dams and individual offspring were recorded on PND 2 (1), and offspring were counted and sex determined. Time mated mice were examined for the number of implantation sites, allowing for calculation of implantation loss. Females that did not give birth or had small litters were killed on PND 3 (1–2) and the dams on PND 22–23 (24–25). Data are expressed as mean ± SEM, offspring data are calculated as litter average.

† Died during instillation.

‡ Weight before exposure.

§ Females in litter (%).

### Organ weights

*Inhalation.* A higher relative brain weight was found in time-mated mice 5 days after exposure to Printex 90 by inhalation, compared to their controls (exposed 2.09 ± 0.04% vs. control 1.86±0.06%, *p*=0.005). Atweaning, relative lung weight was higher in exposed compared to control dams (exposed 1.20 ± 0.03% vs. control 1.10 ± 0.02%, *p* = 0.005). Other organs did not differ.

Organ weights in offspring of dams exposed to Printex 90 were similar to their controls, except that the exposed female offspring had smaller relative heart weight at weaning (exposed 0.68 ± 0.01% vs. control 0.71 ± 0.01%,£= 0.052). Furthermore, exposed adolescent males had a higher relative weight of testes (exposed 2.10 ± 0.05% vs. controls 1.58 ± 0.20%, *p =* 0.024).

*Instillation.* The thymus weight of exposed newborn and weaned offspring was similar to their controls. Other organs were not weighed.

## Discussion

Printex 90 carbon black is one of the best studied materials in particle toxicology. On one hand, it is considered to be a low-toxicity insoluble material, but on the other hand, it is a potent generator of reactive oxygen species (Jacobsen et al. 2008b), it induces DNA strand breaks and oxidatively generated DNA damage (Jacobsen et al. 2008b, 2009), and it is mutagenic (Jacobsen et al. 2007, 2010). Moreover, carbon black induces tumors in rats (Mohr et al. 2006) and is possibly carcinogenic to humans (Baan et al. 2006). Physically and chemically Printex 90 resembles carbonaceous cores of diesel engine combustion particles. Because it is engineered to have nanosize (i.e., the primary particles are smaller than 100 nm), Printex 90 is therefore a representative of an engineered carbonaceous nanoparticles. Although developmental effects of particulate air pollution have been reported in the offspring of human subjects, very few experimental mechanistic studies are available.

We found that maternal inhalation exposure to Printex 90 induced DNA strand breaks in the liver of time-mated mice and in the offspring even weeks after the end of exposure. There were no changes in the levels of DNA strand breaks in mice intratrache-ally instilled with a similar pulmonary dose. Despite this, we did not observe any gestational or developmental toxicity in the offspring.

## Effects in time-mated mice

The mother is the route of exposure for the offspring exposed to xenobiotics *in utero.* The effects of maternal pulmonary exposure to Printex 90 were assessed at two time points in the time-mated mice. Females with few or no offspring were used to evaluate the early effects of exposure, while dams were examined at the end of lactation, at weaning. To make inhalation and instillation comparable, we estimated the dose deposited in the pulmonary region by inhalation, and instilled a similar dose to the highest of three instilled doses (268 jag/animal). As expected, we observed a massive influx of neutrophils in the BAL fluid of exposed females and dams, which persisted for 24-27 days after the end of exposure. After inhalation of Printex 90, the pulmonary inflammation (by polymorphonuclear neutrophil infiltration) in time-mated mice was of similar magnitude as in the mice instilled with the medium dose. We and others have previously found that instilled particles induce stronger inflammatory responses in the lung compared to inhaled particles (Osier and Oberdorster 1997; Driscoll et al. 2000; Jacobsen et al. 2009). This may be because a greater fraction of the instilled particles is deposited deeper into the lung and, consequently, is cleared more slowly.

Generally, translocation of nanoparticles from the lung into circulation is considered to be slow, and it has been reported that only a fraction of a percent gets beyond the lung cavity and regional lymph nodes (van Ravenzwaay et al. 2008; Kreyling et al. 2009; Sadauskas et al. 2009b). In addition, insoluble nanoparticles do not seem to readily pass over the gastrointestinal mucosa in rodents (Carr et al. 1996; Kreyling et al. 2002, 2009). Once in circulation, the distribution to the fetus also seems to be very small (Takahashi and Matsuoka 1981; Myllynen et al. 2008; Wick et al. 2010). However, it is likely that this differs much depending on size, surface and other properties.

We observed DNA strand breaks in liver cells of the exposed time-mated mice and the offspring after inhalation exposure. The exposure procedure is a key determinant for particle size-distribution and consequently for deposition and uptake (Landsiedel et al. 2008). Most Printex 90 particles that would reach the circulation are expected to accumulate in the liver with possible ROS-induced primary genotoxicity. Nanoparticles may persist in the Kupffer cells of the liver for months (Oberdorster et al. 2002; Sadauskas et al. 2007, 2009a). Consequently, only a few liver cells would be directly exposed to ROS generated from Printex 90. Pulmonary exposure to Printex 90 resulted in pulmonary production of cyto-kines (Saber et al. 2005), but no liver inflammation or acute phase response was found in liver after four consecutive nose-only inhalation exposures to Printex 90 or diesel exhaust particles (NIST) (Saber et al. 2009). Thus, it is unlikely that the observed DNA strand breaks are caused by liver inflammation induced by pulmonary exposure. It is also unlikely that the DNA strand breaks were caused by circulating cyto-kines, because we observed the strongest pulmonary inflammation in instillation exposed mice. Inhalation exposure results in a larger immediate exposure to the gastrointestinal tract, because inhaled nanoparticles deposited in the extra-pulmonary region are transported up by mucociliary transport and swallowed. The time-mated mice were exposed by whole-body inhalation exposure and therefore it can be expected that they received even a greater dose in the gastrointestinal tract due to fur grooming. Intra-gastric exposure to 0.64 mg/kg Printex 90 induced DNA damage in the liver of rats 24 h after exposure, whereas the same dose administered by intratracheal instillation caused no DNA damage in the liver or lung (Danielsen et al. 2010). Similarly, intra-gastric administration of other carbonaceous nanoparticles (such as single-wall carbon nanotubes, C_60_ fullerenes and diesel exhaust particles) at the same or even lower doses, caused DNA base oxidation damage in the liver and lung of rats (Danielsen et al. 2008; Folkmann et al. 2009). Therefore, the observed DNA damage in the liver may be a result of the inhalation-associated gastrointestinal exposure rather than from exposure in the lungs.

## Effects in the offspring

The background level of DNA strand breaks was higher in newborns compared to older siblings. These DNA strand breaks might be related to a high proliferation rate during tissue maturation and/or the naturally occurring high level of oxidative stress at birth (Randerath et al. 1996; Cindrova-Davies et al. 2007; McArt et al. 2010). This may have reduced the sensitivity of the comet assay to detect differences between the exposure groups.

A few molecular genotoxins have been demonstrated to pass from the mother to the fetus and generate DNA damage in fetal tissues (Brunborg et al. 1996; Tripathi et al. 2008). However, we expect that only a small fraction of Printex 90 particles can translocate from the lungs of the mothers to the fetuses because the particles would have to pass two compartmental barriers, i.e., in the lung and placenta. The observed effects of *in utero* exposure are therefore more likely due to changes in signalling cascades. It is possible that inflammatory molecules are transferred from the maternal to the fetal compartment (Jonakait 2007) and affect the fetus. Thus, the increased levels of DNA strand breaks in liver tissue of the offspring may be caused by maternally induced inflammatory mediators after Printex 90 inhalation exposure.

DNA strand breaks in offspring liver of the inhalation exposed dams were still evident in 50-day old offspring. At this time, the offspring were independently fed and had no contact with the dams. Therefore, it is unlikely that secondary genotoxicity caused by inflammatory signalling from the dams caused the observed DNA strand breaks in the older offspring.

Neither inhalation nor instillation of Printex 90 affected gestational or lactational parameters, and offspring of exposed dams survived and developed similarly to control offspring. This is in agreement with findings in two other published studies; an instillation study of carbon nanoparticles (200 jag/mouse on gestational days 7 and 14) (Yoshida et al. 2010) and a study of TiO_2_ from our laboratory using a set-up similar to the present inhalation exposure (Hougaard et al. 2010), suggesting that these inhaled nanosize particles are not toxic during development. Human exposure to air pollution has been associated with adverse effects *in utero* exhibited by reduced growth, increased mortality and increased risk of perinatal diseases (Dejmek et al. 1999; Lacasana et al. 2005; Sram et al. 2005; Slama et al. 2007; Brauer et al. 2008; Pedersen et al. 2009; Pope et al. 2010). Children born and raised in areas with high air pollution have systemic inflammation and increased levels of urinary 8-oxodeoxyguanosine, a marker of oxidative damage to DNA (Calderon-Garciduenas et al. 2008; Svecova et al. 2009). Our data indicate that inhalation exposure to carbon black Printex 90 may have long-lasting genotoxic effects on the exposed organism.
